# Supplementary figures and images for: Laboratory Experiments Suggest a Limited Impact of Increased Nitrogen Deposition on Snow Algae Blooms
Source: Environ Microbiol Rep. 2024 Nov 28;16(6):e70052. doi: 10.1111/1758-2229.70052 (PMC11604572; doi:10.1111/1758-2229.70052)

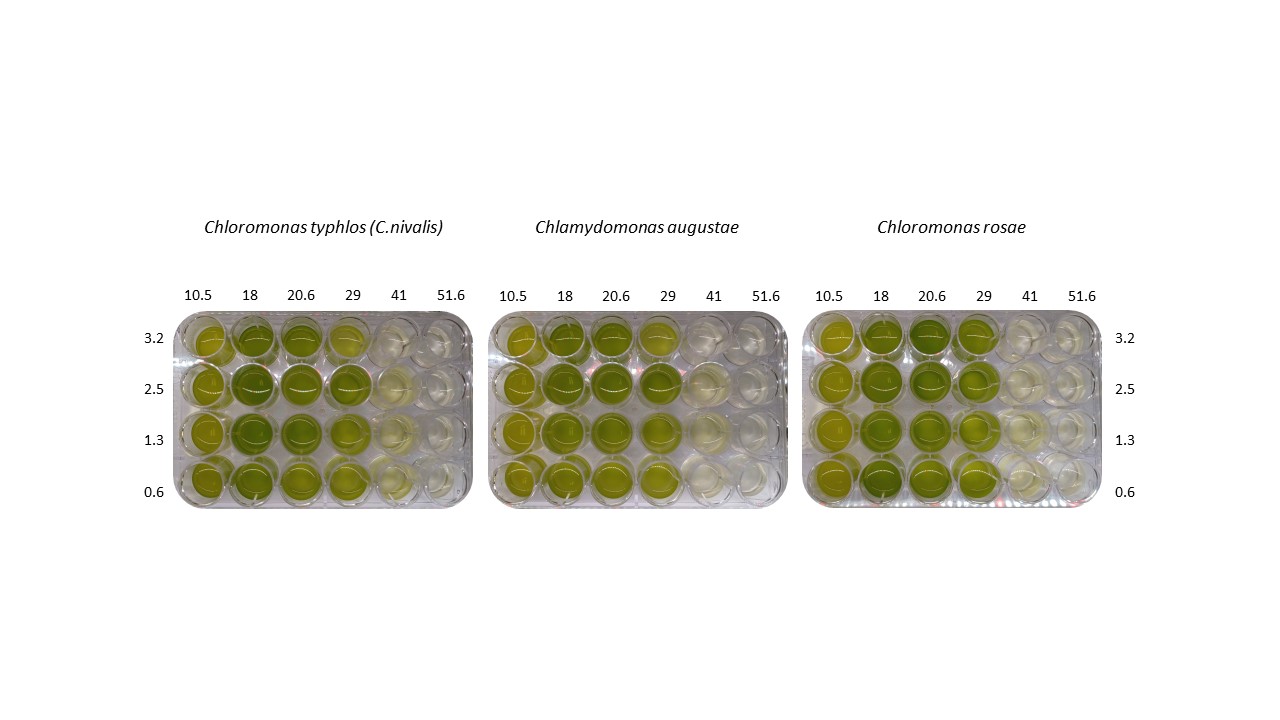

Supplement: Supplementary file 2 — Figure S1. Images of the 24‐well plates showing the three snow algae strains used in this experiment at the end of the experiment, 38 days after the start. [file EMI4-16-e70052-s001.jpg]

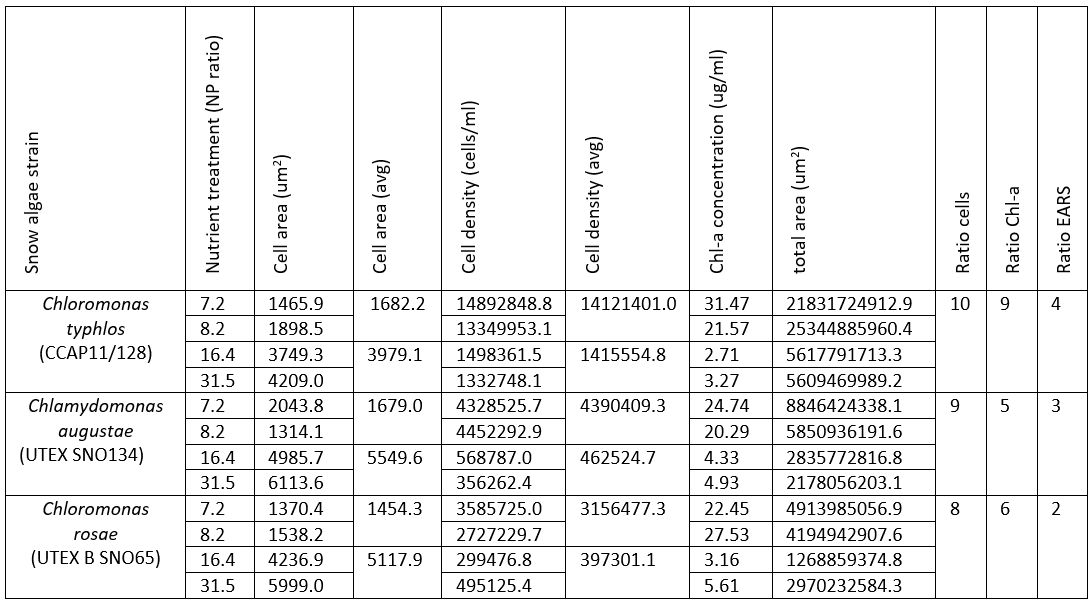

Supplement: Supplementary file 4 — Table S2. Summary of cell area, cell density, total area (EARS), total chlorophyll‐a concentrations and ratios of cell densities, chlorophyll‐a concentrations and EARS across the selected nutrient treatments for the three snow algae strains. [file EMI4-16-e70052-s004.docx]
