# Supplementary material for: Laboratory Experiments Suggest a Limited Impact of Increased Nitrogen Deposition on Snow Algae Blooms
Source: Environ Microbiol Rep. 2024 Nov 28;16(6):e70052. doi: 10.1111/1758-2229.70052 (PMC11604572; doi:10.1111/1758-2229.70052)
Supplement: Supplementary file 3 — Table S1. Experimental design with N and P supply (μmol L−1, bold letters) and molar nutrient ratios for all 24 combinations of N and P supply. [file EMI4-16-e70052-s002.docx]

|  | N (µmol L^-1^) | | | | |
| --- | --- | --- | --- | --- | --- |
| P (µmol L^-1^) | **10.5** | **18.0** | **20.6** | **29.0** | **41.0** |
| **3.2** | 3.3 | 5.6 | 6.4 | 9.1 | 12.8 |
| **2.5** | 4.2 | 7.2 | 8.2 | 11.6 | 16.4 |
| **1.3** | 8.1 | 13.8 | 15.8 | 22.3 | 31.5 |
| **0.6** | 17.5 | 30.0 | 34.3 | 48.3 | 68.3 |
